# Epithelioid Trophoblastic Tumor: A Case Report and Review of the Literature

**DOI:** 10.1155/2012/862472

**Published:** 2012-12-02

**Authors:** Eirwen M. Scott, Ashlee L. Smith, Mohamed Mokhtar Desouki, Alexander B. Olawaiye

**Affiliations:** ^1^Department of Gynecologic Oncology, Magee-Womens Hospital of UPMC, 300 Halket Street, Pittsburgh, PA 15213, USA; ^2^Department of Pathology, Breast, and Gynecologic Pathology, Magee-Womens Hospital of UPMC, 300 Halket Street, Pittsburgh, PA 15213, USA

## Abstract

Epithelioid trophoblastic tumor (ETT) is a rare gestational trophoblastic tumor. Cases of ETT present as abnormal vaginal bleeding in women of reproductive age, with low human chorionic gonadotropin (hCG) levels. ETT can be a sequela of any gestational event and can present in both intrauterine and extrauterine sites. Metastasis and death have been reported. We present a case of a 44-year-old female incidentally diagnosed with ETT following laparoscopic-assisted vaginal hysterectomy. Postoperative evaluation for metastatic disease was negative. The patient has been closely followed and remains disease free 8 months postoperatively. ETT presents a diagnostic challenge due to its rarity and histologic resemblance to other pathologies. ETT is relatively chemoresistant and managed surgically. Misdiagnosis delays effective treatment and affects survival.

## 1. Introduction

Gestational trophoblastic disease is defined by abnormal proliferation of placental trophoblasts. It can be further classified into benign and malignant lesions. Benign lesions include placental site nodule, exaggerated placental site, and hydatidiform moles. Malignant lesions, termed gestational trophoblastic neoplasia, include choriocarcinoma (CC), placental site trophoblastic tumor (PSTT), epithelioid trophoblastic tumor (ETT), and invasive moles that do not spontaneously resolve. ETT, first described by Shih and Kurman in 1998, is a rare gestational trophoblastic tumor arising from intermediate trophoblastic cells of the chorionic laeve [1]. Often misdiagnosed as CC, PSTT, or cervical squamous cell carcinoma, ETT is characterized by specific histologic and immunophenotypic patterns.

In 2008, Palmer et al. reviewed the existing literature, identifying 19 English papers and 52 cases of epithelioid trophoblastic tumor diagnosed from 1989 to 2007 [2]. A MEDLINE search identified 7 additional publications, reporting 9 cases that have since been published. Non-English publications, including 2 French cases, 6 Chinese cases, and 25 Czech cases, were excluded due to lack of access to translation resources. To the best of our knowledge, 94 reported cases of ETT have been identified to date. We present the case of a 44 -year-old female incidentally diagnosed with ETT following laparoscopic assisted vaginal hysterectomy for pelvic organ prolapse. 

## 2. Case Report

A 44-year-old gravida 2, para 2002, presented to her primary gynecologist with complaints of pelvic pressure and discomfort. Her obstetric history was significant for 2 uncomplicated full term spontaneous vaginal deliveries, in 2006 and 2008. She denied intermenstrual bleeding, post-coital bleeding, and recent changes in her menstrual cycle. Her physical exam was notable only for a large cystocele, small rectocele, second-degree uterine prolapse, and a left ovarian cyst. Pap smear completed prior to the surgery was normal. The patient underwent an uncomplicated laparoscopic-assisted vaginal hysterectomy, left salpingo-oophorectomy, right salpingectomy, and anterior and posterior colporrhaphy. Her serum quantitative hCG on the day of surgery was negative. Epithelioid trophoblastic tumor of the lower uterine segment and endocervix was incidentally discovered on tissue pathology. 

**Figure 1:**
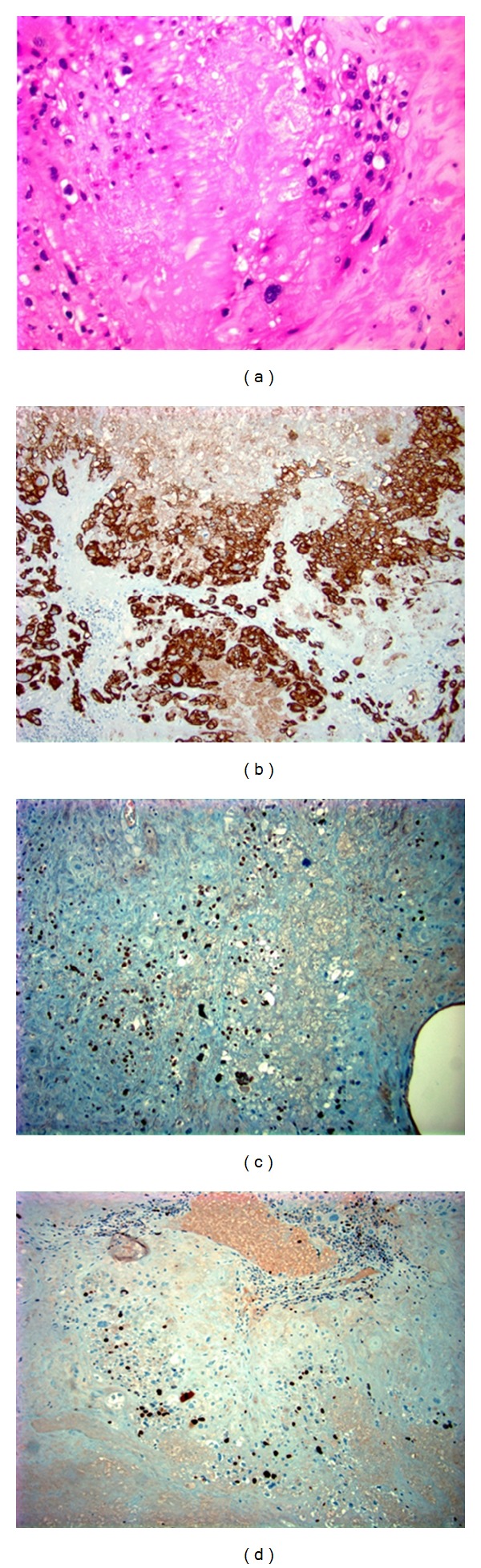
Pathologic evaluation of hysterectomy specimen. (a) H&E staining; (b) Pankeratin AE1/AE3 staining; (c) p63 staining; (d) ki67 staining.

Grossly, the uterus measured 9.5 × 6.5 × 4.0 cm, with a slightly puckered area near the anterior flap of the serosal surface. The endocervical canal was tan and rugous with a well-defined squamocolumnar junction. Within the anterior aspect of the lower uterine segment, there was a 2.5 × 2.4 × 0.9 cm area of tan-white, friable tissue. A minimal amount of friable tissue extended laterally onto the posterior aspect of the lower uterine segment. The remainder of the uterus appeared grossly normal. The left ovary measured 5.8 × 4.8 × 2.8 cm with a smooth, pink-tan surface. The specimen was sectioned to reveal a biloculated, cystic structure measuring 4.0 × 3.5 × 2.5 cm with no excrescences or solid areas. No gross lesions were identified in the fallopian tubes. The specimen margins were, notably, free of tumor. Immunoperoxidase staining of the trophoblastic cells was positive for pancytokeratin AE1/AE3, p63, EMA, CD10, inhibin alpha, and Ki67 (Figure 1). The cytology of pelvic washings was negative for malignant cells. 

The patient was referred to and seen in our gynecologic oncology division at Magee-Womens Hospital of UPMC 2 weeks post-operatively. A repeat qualitative serum hCG was negative, as was human placental lactogen (hPL). Chest X-ray and CT of the chest, abdomen, and pelvis were negative, with the exception of a 4 × 4.6 cm complex right para-rectal mass, presumed to be the patient's right ovary. The mass was further characterized by pelvic ultrasound. Eight months post-operatively, the patient remains disease free. She is followed monthly with serum hCG and hPL. 

## 3. Discussion

In 1998, Shih and Kurman described epithelioid trophoblastic tumor (ETT) as a diagnosis distinct from placental site trophoblastic tumor (PSTT) and choriocarcinoma (CC). Initially termed “atypical choriocarcinoma,” ETT histology was first described in pulmonary lesions in patients undergoing chemotherapy for CC [3, 4]. Similar histology was subsequently reported for intrauterine lesions [5]. Until 1994, these lesions had only been described in patients with a history of chemotherapy for gestational trophoblastic disease (GTD), suggesting that the atypical tumor developed as a result of an inadequate response of the antecedent choriocarcinoma or hydatidiform mole to chemotherapy. Hypotheses suggested that either chemotherapy prolonged the course of GTD allowing the atypical growth pattern to develop or that chemotherapy directly induced tumor cell alterations [4]. 

### 3.1. Clinical Presentation

Shih and Kurman published a review of 14 cases of epithelioid trophoblastic tumor in patients with no antecedent history of chemotherapy for GTD. They described ETT, along with its characteristic histologic and immunohistochemical patterns, as an entity distinct from other forms of trophoblastic disease [1]. While the vast majority of cases have been reported in women of reproductive age, one case described a 66-year-old postmenopausal woman with ETT [6]. ETT can present as isolated uterine/cervical disease, as isolated extrauterine disease, or as a primary uterine tumor with metastasis. Most often, the uterus is the primary site of ETT (40%), followed by the cervix (31%). The lung is the most common extrauterine site, accounting for 19% of cases [1, 2, 7, 8]. Other cases of extrauterine disease have been reported in the small bowel, vagina, fallopian tube, broad ligament, and gallbladder [1, 9–11]. Sixty-seven percent of patients with ETT present with abnormal vaginal bleedingand 25–35% present with metastasis, most frequently of the lung [1, 2]. In our case, ETT was incidentally found in the pathology specimen of an asymptomatic female undergoing hysterectomy for pelvic organ prolapsed. To our knowledge, the only other case of asymptomatic ETT was identified in the D&C pathology of a patient undergoing evaluation of ectopic pregnancy [1]. Most commonly associated with prior term deliveries (43%), ETT has also been associated with molar pregnancies (39%), and abortions (18%), occurring 2 to 300 months (mean 76) after the antecedent gestational event [1, 2]. As reported by Palmer et al., both intrauterine and extrauterine ETT present with elevated hCG levels, though in 69% of cases hCG was less than 2500 [2]. Infrequently, hCG testing is negative, as in our case. Only 5 previously reported cases of ETT report negative hCG.

### 3.2. Histology

Grossly, epithelioid trophoblastic tumor appears as a discrete, nodular, expansile lesion with solid, cystic, and hemorrhagic components. Histologically, ETT is composed of nests of uniform mononucleate chorionic type intermediate trophoblastic cells, with eosinophilic or clear cytoplasm, round nuclei, and a well-defined cell membrane. Nests of trophoblastic cells are surrounded by extensive necrosis and a hyaline like matrix, resembling keratin, giving ETT its characteristic “geographic” appearance. Within the center of each tumor nest, there is often a small blood vessel, though overall the tumor lacks significant vascular invasion. ETT can be histologically distinguished from placental site trophoblastic tumor (PSTT), a tumor of implantation type intermediate trophoblastic cells. Intermediate cells of PSTT are larger, have more nuclear pleomorphism, and have a more infiltrative growth pattern and vascular invasion. Placental site nodules (PSN) have a more benign appearance than ETT in so much as they are less cellular, less necrotic, and display less nuclear atypia. Due to its similar, yet more malignant, appearance, ETT is hypothesized to represent the malignant counterpart to PSN [2]. Choriocarcinoma (CC) is easily distinguished from ETT by its dimorphic trophoblastic cell population (cytotrophoblasts, syncytiotrophoblasts). Additionally, CC is associated with significant hemorrhage, not characteristically present in ETT. The well-circumscribed, nodular growth pattern of ETT is not seen in CC. The hyaline like matrix and necrosis present in ETT can resemble keratin and has reportedly lead to the misdiagnosis of cervical squamous cell carcinoma (SCC) [12–16]. In addition to keratin, SCC is further marked by the presence of keratin pearls and intercellular bridges [13].

### 3.3. Immunohistochemical Staining

Epithelioid trophoblastic tumor (ETT) also has a characteristic immunohistochemical staining pattern. It is often diffusely positive for inhibin alpha (a common marker of trophoblastic lesions), cytokeratin AE1/AE3, epithelial membrane antigen, E-cadherin, prolyl 4-hydroxylase, and epidermal growth factor receptor. Conversely, it is often only focally positive for trophoblastic proteins including HPL, HCG, P1AP, and MelCAM. Placental site trophoblastic tumor (PSTT) stains diffusely positive for hPL, P1AP, and MelCAM. CC stains diffusely positive for hCG and less strongly for hPL. Epithelioid leiomyosarcoma is easily differentiated from trophoblastic tumors by immunohistochemical staining for smooth muscle markers. Inhibin alpha and cytokeratin 18 are particularly helpful in distinguishing ETT from cervical squamous cell carcinoma (SCC), which does not express these proteins. 

Recent studies have examined the role p63 staining in distinguishing ETT from common misdiagnoses. Results indicate that p63 is found in chorionic type intermediate trophoblastic cells (as in ETT, PSN), but not from implantation type intermediate trophoblastic cells (as in PSTT) [17, 18]. p63 can help to distinguish ETT from PSTT, however, does not differentiate ETT from cervical SCC [19]. Another recent study looked at the roles of cyclin E and p16 in distinguishing ETT, PSN, and cervical SCC. Results showed significant cyclin E expression in ETT, less evident in PSN, and diffuse p16 immunoreactivity in cervical SCC, absent in ETT and PSN [20]. Algorithms based on immunophenotypic patterns have been developed to differentiate trophoblastic lesions [18, 21].

Ki67 nuclear labeling indices have also been studied and may help differentiate ETT from other trophoblastic tumors and SCC. Shih reported, in 1998, a mean Ki67 labeling index of 17.7% (range 10–25%) for ETT. Conversely, PSN, CC, and cervical SCC have much higher labeling indices [1, 12]. The presence of Y chromosomal material in PSTT and ETT provides molecular evidence of the trophoblastic origin of ETT and differentiates trophoblastic tumors from SCC [14, 22]. Though immunohistochemical markers help to distinguish varying trophoblastic tumors from one another, authors have reported cases of coexisting trophoblastic tumors, further complicating the diagnostic process [23].

### 3.4. Management

Due to the rarity of disease, epithelioid trophoblastic tumor remains a diagnostic challenge despite intense research efforts to elucidate its phenotypic patterns. Appropriately identifying cases of ETT is a critical component of treatment planning. Whereas choriocarcinoma is chemosensitive, ETT is relatively chemoresistant. Consequently, surgical resection remains the primary treatment modality. Palmer et al. summarize treatment strategies employed in 52 reported cases of ETT. Thirty-nine percent of patients were treated with surgery only (31% TAH, 4% D&C, 4% lung resection). In total, surgical intervention included hysterectomy (73%), D&C (19%), lung resection (21%), bowel resection (2%), and wide local excision of vaginal tumor (2%). Four percent of patients underwent radiation therapy. Twenty-nine percent of patients had preoperative chemotherapy and 48% had chemotherapy post-operatively, though specific regimens were quite variable. First line chemotherapy agents used, in various combinations, included methotrexate (+/− leucovorin), actinomycin, adriamycin, cytoxan, cisplatin, vincristine, hydroxyurea, dactinomycin, melphelan, etoposide, and 5-fluorouracil. Methotrexate, actinomycin, and chlorambucil (MAC) and etoposide, methotrexate, actinomycin D, cyclophosphamide, and oncovin (EMACO) were 2 relatively common regimens. Unfortunately, due to the significant variability of chemotherapy regimens and lack of standardization of therapy, it is difficult to draw conclusions regarding chemotherapy response rates. 

For patients with intrauterine disease, hysterectomy is critical for both diagnostic and therapeutic purposes [2]. Extrauterine disease, when possible, is also preferentially managed surgically, that is, lung resection or bowel resection [1, 2]. Lewin et al. reported 3 cases of isolated pulmonary lesions and elevated hCG, with no evidence of uterine disease. All 3 patients underwent lung resection and hysterectomy and pathology reports supported the diagnosis of isolated pulmonary ETT. While only 2 patients had adjunctive chemotherapy, all 3 are alive without evidence of disease following surgical resection of the tumor [7]. With the accurate diagnosis of ETT, chemotherapy has largely been reserved for treatment of metastatic, recurrent, or surgically unresectable disease. Resection of isolated metastasis is recommended, when feasible. 

Because appropriate treatment differs for epithelioid trophoblastic tumor, choriocarcinoma, and cervical squamous cell carcinoma, establishing an accurate diagnosis prevents delays in care. Jordan et al. report a case of ETT that presented as a cervical mass and elevated hCG 18 months after a spontaneous abortion. Initially, the patient was diagnosed with coexisting stage IIIB squamous cell carcinoma of the cervix and gestational trophoblastic neoplasia and treated with radiosensitizing cisplatin and pelvic radiation, in addition to methotrexate. When her disease progressed to pelvic lymph nodes despite conventional therapy, her tumor pathology was reviewed and diagnosis changed to ETT. Despite multiple chemotherapy regimens, her disease progressed and she died from disease 24 months after diagnosis. The authors maintain that surgical management was the optimal treatment strategy for this patient with ETT and that the delay in diagnosis altered the management [15]. Similarly, Shet et al. report a case of ETT initially diagnosed as CC and treated with chemotherapy (EMACO). She subsequently underwent surgical excision of an adnexal and uterine mass and pathology reported the tumor to be ETT. Despite multiple chemotherapy regimens, the patient's disease progressed and decision was made to proceed with palliative care. Again, the misdiagnosis of CC leads to insufficient treatment, lacking surgical intervention [24].

Death rates of 10–13% have been reported for epithelioid trophoblastic tumor [2]. Identifying prognostic factors is challenging given the absence of long-term follow up data on reported cases. Takekawa et al. suggest that prognostic factors of ETT may be similar to those of PSTT [25]. Poor prognostic factors for PSTT include tumor extension beyond the uterus, age >40, interval from prior pregnancy >2 years, and mitotic counts >5/10 per HPF [26].Others propose that high mitotic indices and Ki67 nuclear labeling indices are associated with malignant behavior [12]. However, in reported cases of ETT with unusually high Ki67 nuclear labeling indices, both patients are alive and well postoperatively though neither patient presented with metastases and both were treated solely with surgical intervention [6, 13]. In a review of nine patients with ETT, Shen et al. identified multifocal lesions in bulky uterus, full-thickness myometrial invasion, and uterine serosal involvement as risk factors which could be linked to poor outcomes in these patients [27].

Epithelioid trophoblastic tumor is a rare gestational trophoblastic tumor with distinct histologic and immunophenotypic patterns. Because of its rarity and large spectrum of clinical presentation, it often goes misdiagnosed, and consequently, mismanaged. Our case presents two unusual features of epithelioid trophoblastic tumor, asymptomatic presentation and negative serum hCG. Additionally, post-operative surveillance for recurrence of this tumor, which presented asymptomatically with negative serum markers, remains a clinical challenge. Limited data has been published regarding follow up and surveillance for recurrence. As such, this case was reviewed by the team of physicians in our gynecologic oncology division at Magee Women's Hospital. A collaborative decision was made to monitor this patient with clinical follow up every 3 months, in addition to monthly serum hCG and hPL. A high clinical suspicion for ETT, and pathologists knowledgeable about its characteristic histologic and immunophenotypic patterns, are essential components of patient care. 

## Consent

Written informed consent was obtained from the patient for publication of this case report and accompanying images. A copy of the written consent is available for review by the Editor-in-Chief of this journal, upon request.
